# Tumor Microenvironment and its Implications for Antitumor Immunity in Cholangiocarcinoma: Future Perspectives for Novel Therapies

**DOI:** 10.7150/ijbs.73949

**Published:** 2022-08-21

**Authors:** Hengsong Cao, Tian Huang, Mingrui Dai, Xiangyi Kong, Hanyuan Liu, Zhiying Zheng, Guoqiang Sun, Guangshun Sun, Dawei Rong, Zehua Jin, Weiwei Tang, Yongxiang Xia

**Affiliations:** 1 Hepatobiliary/Liver Transplantation Center, The First Affiliated Hospital of Nanjing Medical University, Key Laboratory of Living Donor Transplantation, Chinese Academy of Medical Sciences, Nanjing, Jiangsu, China.; 2 Stomatological college of Nanjing Medical University, Nanjing, Jiangsu, China.; 3 Department of General Surgery, Nanjing First Hospital, Nanjing Medical University, Nanjing, Jiangsu, China.; 4 Department of Anesthesiology, The First Affiliated Hospital of Nanjing Medical University, Nanjing, Jiangsu, China.; 5 Department of Gastroenterology, The First Affiliated Hospital of Nanjing Medical University,Nanjing, Jiangsu, China.

**Keywords:** cholangiocarcinoma, tumor microenvironment, immune mechanism, targeted therapy, prognostic markers, immunotherapy

## Abstract

The incidence of cholangiocarcinoma (CCA) has been increasing over the past few years. Although there are surgery, chemotherapy and other conventional treatment methods, the effect is not as expected. At present, immunotherapy has become the research frontier of cancer treatment, and CCA tumor microenvironment (TME) is becoming a hot exploration direction of immunobiology. TME can affect tumor progression through changes in metabolism, secretion and immunity. Accordingly, understanding the role played by immune cells and stromal cells in TME is important for the study of CCA immunotherapy. This review will discuss the interactions between immune cells (including CD8^+^ T cells, CD4^+^ T cells, macrophages, natural killer cells, dendritic cells, myeloid suppressor cells, mast cells, and neutrophils) and stromal cells (including cancer-associated fibroblasts, endothelial cells) in the TME of CCA. In addition, we will also discuss current research results on TME of CCA and recent advances in immunotherapy.

## Introduction

**Figure 1:**
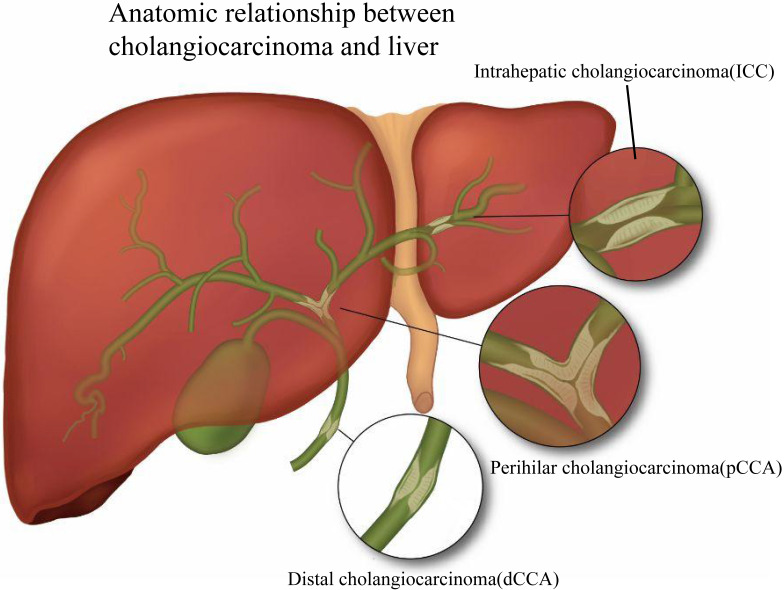
According to the relative anatomical location with the liver, CCA is divided into three types: ICC, pCCA and dCCA. ICC is defined as bile duct cancer located inside the liver, and the histological types are classified as mass-forming, periductal infiltrating, and intraductal growing. pCCA is defined as a secondary branch located in the hepatic duct (from the common bile duct above the cystic duct to the liver) and histologically as a Klatskin tumor. dCCA refers to distal cholangiocarcinoma located extrahepatically, and the histological type is mainly adenocarcinoma.

Cholangiocarcinoma (CCA) is an epithelial malignant tumor originating from the biliary tract and is the second most common primary liver malignancy after hepatocellular carcinoma 1. Based on anatomical site, CCA is divided into intrahepatic cholangiocarcinoma (ICC), perihilar cholangiocarcinoma (pCCA), as well as distal cholangiocarcinoma (dCCA) 2. Fig. 1 presents the classification of CCA based on its relative location to the liver. ICC is malignant tumors caused by the biliary epithelium, and like hepatocellular carcinoma, ICC is usually classified as primary liver cancer, and in the review, we classified it into CCA. According to the American NCCN guidelines 3, ICC is classified into three types, including mass-forming, periductal-infiltrating, and intraductal-growing. The definition of pCCA has been controversial, and we believe that the location of pCCA is defined as the secondary branch of the hepatic duct from the common bile duct above the cystic duct to the liver, which is histologically a Klatskin tumor. dCCA is uncontroversial in definition, and it is the carcinoma of the distal extrahepatic bile duct 4. Existing research has suggested that dCCA accounts for approximately 50% of the total number of CCAs, pCCA accounts for nearly 40%, and the rest is ICC 5.

With the change over time in environmental and immunological factors (e.g., the effects of hepatolithiasis or hepatitis virus infection), the incidence of ICC increases. The reported incidence of ICC in the United States increased from 0.44 cases per 100,000 to 1.18 cases per 100,000, with an annual percentage change (APC) of 2.30%. The above trend has accelerated to 4.36% over the past decade 6. Early detection and diagnosis of CCA is still a challenge due to its clinical characteristics, such as deep intrahepatic anatomical location, high fibrous hyperplasia, and few tumor cells. Currently, surgery is considered an effective treatment for CCA. For instance, the 5-year survival rate of patients with early CCA after liver transplantation is as high as 65%, but is limited to early nonmetastatic disease 7. For non-early CCA, recurrence rate after surgical resection exceeds 50% 8. Besides surgical treatment, the OS (overall survival) of chemotherapy drugs such as GEMCIS is only 11.7 months 9. Compared with other cancers (e.g., hepatocellular carcinoma), CCA is less effective in chemotherapy and has an unsatisfactory prognosis. Thus, molecular targets and effective therapeutic measures for the treatment of CCA are urgently required.

**Figure 2:**
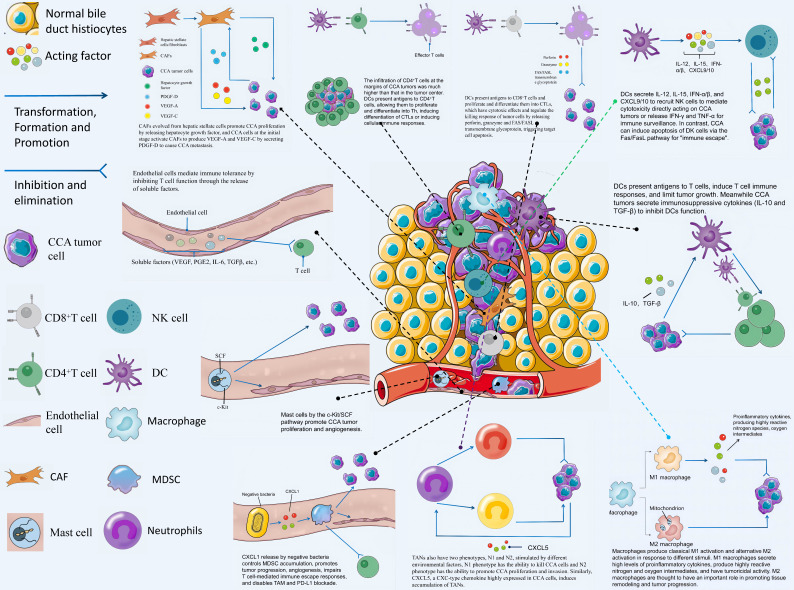
CCA TME refers to an intrinsic environment for the interaction between various cells and tumor cells including CD4 ^+^ T cells, CD8 ^+^ T cells, Tregs, DCs, NK cells, vascular endothelial cells, macrophages, neutrophils, CAFs, mast cells and MDSCs as well as body tissues.

Tumor microenvironment (TME) has been increasingly reported over the past few years, opening a new approach for immunotherapy targeting CCA. TME is an ever-changing dynamic heterogeneous collection of tumor cells, infiltrating and hosting host cells, secretory factors, and extracellular matrix 10. It includes not only the structure, function, and metabolism of the tissue where the tumor is located, but also the internal environment of the tumor cell itself (nucleus and cytoplasm), promoting the occurrence and development of the tumor. The TME of CCA is mainly composed of immune-related cells, tumor cells and tumor stromal cells 11. Thanks to the development of technologies such as single-cell sequencing, research on immune-related cells in CCA TME has promoted the development of CCA immunotherapy. Based on the application of immunosuppressive agents in the clinical treatment of HCC, many immunotherapy clinical trials on CCA are also being carried out successively and have achieved some results. For instance, immunosuppressive agents alone for the treatment of advanced BTC 12 or immunosuppressive agents in combination with chemotherapeutic drugs 13. A phase II study of lenvatinib plus PD-1 inhibitor as first-line treatment for patients with unresectable BTC showed an encouraging result with mOS (median overall survival) of 17.7 months 14. In this study, we will review the various components of TME and their mechanisms of action on CCA tumors, and highlight the important role played by TME in immunotherapy for CCA. Fig. 2 contains a schematic representation of the interactions of each component of CCA TME.

## CCA-associated tumor stromal cells in CCA

Cancer is a systemic disease that consists of multiple components of the tumor cells and host stromal cells. It has been proven that stromal cells in the TME play a vital role in cancer development. It is possible to define the molecular events by which reactive stromal cells affect cancer cells and thus to identify biomarkers and therapeutic targets. In general, TME stromal cells comprise endothelial cells in blood and lymph, stem cells, as well as tumor-associated fibroblasts 15. Vascular endothelial cells and cancer-associated fibroblasts (CAFs) take on a great significance in CCA.

### CAFs in CCA

Among the tumor stromal cells involved in CCA TME, CAF mechanisms are the most abundant and essential for driving tumor proliferation, which are investigated in this study. Unlike normal fibroblasts, CAFs are the most abundant source and the major components of tumor stroma and secreted growth factors, inflammatory ligands, and extracellular matrix (ECM) proteins 16. Thanks to the development of RNA single-cell sequencing technology, we can gain insights into the heterogeneity of CAFs with normal fibroblasts and its mechanism of action. The role played by CAFs in the TME of CCA is not single, the interior of CAFs is a heterogeneous collection of cell populations, and CAFs can either facilitate CCA development or inhibit CCA. CAFs from hepatic stellate cells (HSCs) are capable of mediating the release of hepatocyte growth factor from inflammatory CAFs through the direct interaction of the HSC-CAF-tumor pathway that facilitates the proliferation of ICCs via tumor-expressed MET (mesenchymal to epithelial transition factor) 17. Zhang et al. have suggested that IL-6 (interleukin-6) secretion by vCAFs (vascular cancer-associated fibroblasts) significantly enhances the malignancy of ICC cells through IL-6/IL-6R axis interaction between CAFs and tumor cells, while exosomal miR-95p derived from ICC cells can induce IL-6 expression in vCAFs 18. However, high expression of miR-34c, another tumor-derived exosome, can target and inhibit Wnt-1 to activate the Wnt signaling pathway in CCA and inhibit the conversion of fibrocytes into CAFs 19. It is necessary to conduct in-depth research on the mechanism of action of different exosomes on CAFs in the TME of CCA. At the initial stage of ICC, ICC cells recruit and activate CAF to stimulate fibroblasts to produce VEGF-C and VEGF-A by secreting PDGF-D, resulting in lymphangiectasis and tumor cell injection, which causes tumor metastasis 20. Another study has suggested that secretion of FAP (fibroblast activation protein) by CAFs activates the FAP-STAT3-CCL2 signaling pathway to facilitate connective tissue proliferation in ICC 21.

**Figure 3:**
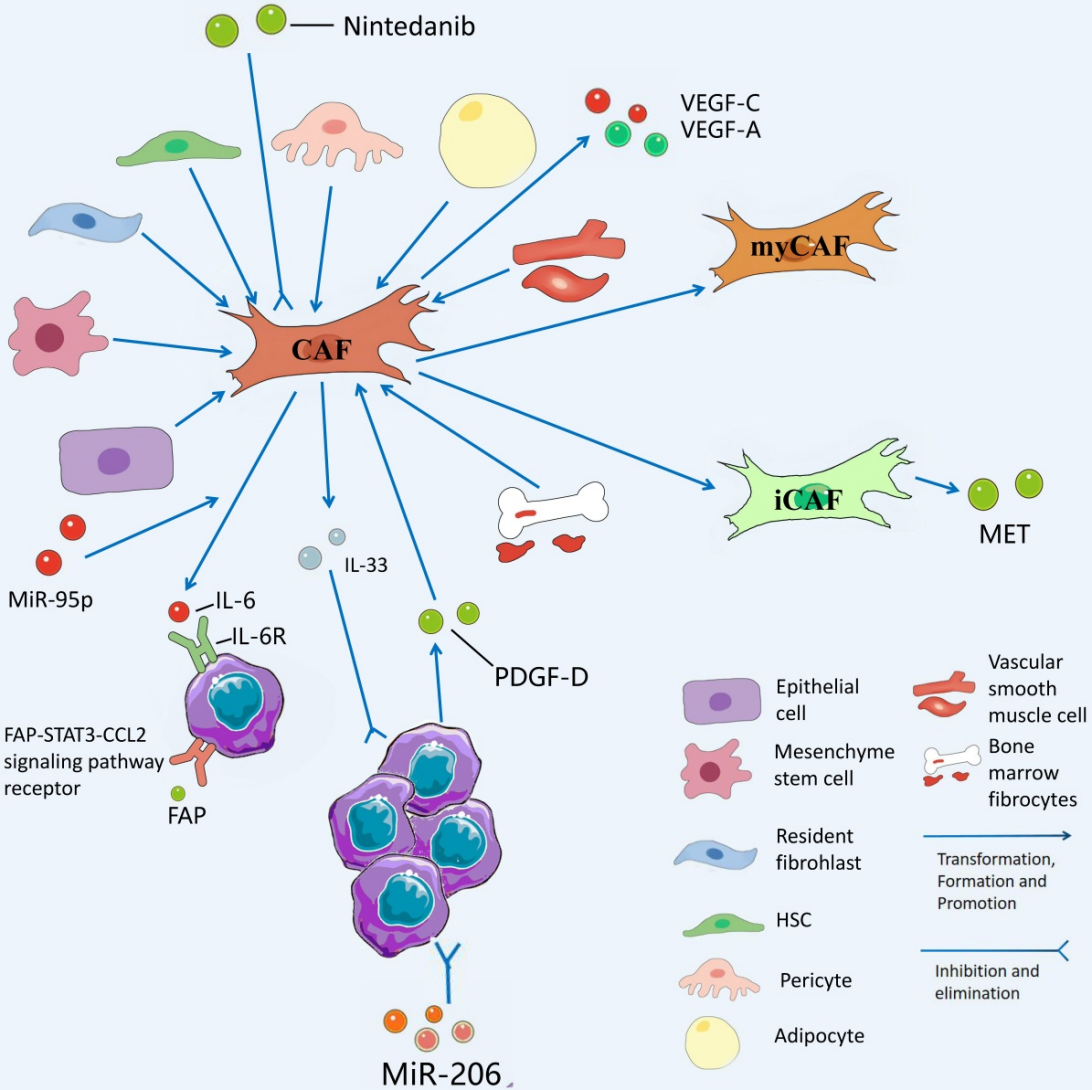
CAFs has diverse sources and is differentiated from epithelial cells, mesenchyme stem cells, resident fibrohlast, HSCs, pericyte, adipocyte, vascular smooth muscle cells, and bone marrow fibrocytes. CAFs from hepatic stellate cells (HSCs) mediate the release of hepatocyte growth factor from inflammatory CAFs through direct interaction of the hsc-cafa-tumor pathway and promote the proliferation of ICC through tumor-expressed MET. VCAFs (vascular carcinoma-associated fibroblasts) secrete IL-6 (interleukin-6) to enhance the malignancy of ICC cells through the interaction of the IL-6/IL-6R axis with tumor cells, while exosomal miR-95p of ICC cells can induce IL-6 expression in vCAFs. High expression of miR-34c in tumor-derived exosomes can target and inhibit Wnt1, allowing it to activate the Wnt signaling pathway in CCA and slow the conversion of fibrocytes to CAFs. Nintedanib can treat refractory CCA by inhibiting the activation of CAFs.

CAFs and their secreted products play a role in inhibiting the proliferation of CCA. Some studies have suggested that the prognosis of patients with high IL-33 content in CAFs in CCA patients is significantly better than that of patients with low IL-33 content in CAFs, suggesting that IL-33 is a marker of good prognosis in CCA and strengthening IL-33 in CAFs is a promising therapeutic direction 22. Moreover, the interaction between CCA and CAFs to facilitate tissue proliferation and tumor metastasis provides a novel entry point for targeting CAFs to treat CCA. For instance, Takahiro et al. suggested that nintedanib is capable of treating refractory ICC by inhibiting the activation of CAFs after the use of nintedanib 23. It has also been confirmed that MiR-206 inhibits the deterioration of ICC and increases chemosensitivity by inhibiting the interaction with interstitial CAFs 24. Table 1 lists the promoting and inhibiting effects of CAFs on CCA. Fig. 3 presents the role played by CAFs in CCA TME.

### Endothelial cells in CCA

Tumor blood vessels primarily comprise endothelial cells, which are critical to cancer progression and provide essential nutrients for tumor growth. Tumor-associated endothelial cells can facilitate tumor progression and metastasis 25. eNOS (endothelial Nitric Oxide Lyase) expressed in endothelial cells significantly affects the metastasis and angiogenesis of CCA^26^. In addition, endothelial cells mediate immune tolerance through the unbalanced expression of surface molecules and the release of soluble factors (e.g., VEGF, PGE-2, IL-6, TGF-β) that inhibit T cell function 27. In the tumor microenvironment, CCA cells DKK1 (paracrine dickkopf-related protein 1) to enhance the angiogenic potential of tumor-associated endothelial cells 28. VEGFR1 and VEGFR2 are critical receptors in the process of angiogenesis. Xu et al. suggested that HMGB-1 (high mobility group box 1) released by perihilar CCA cells is capable of up-regulating the expression of VEGFR2 in vascular endothelial cells 29. ERK5 silencing down-regulates the expression of angiopoietin-1 and VEGF in the CCA tumor microenvironment to induce angiogenesis, which reduces the ability of HUVEC (human umbilical veins) 30. Circ-CCAC1 in CCA-derived extracellular vesicles can disrupt endothelial cell barrier integrity and induce angiogenesis in tumor tissues 31. Besides the formation of blood vessels, endothelial cells also play a role in the formation of lymphatic vessels in tumor tissues. Cadamuro et al. suggested that PDGF-D from ICC recruits lymphatic endothelial cells (LECs) to aggregate by stimulating paracrine signals produced by CAFs to facilitate the formation of tumor-associated lymphatic vessels 20. Therefore, the current research hotspot is to explore the targets and methods of inhibiting tumor angiogenesis. Ramucirumab is a fully human immunoglobulin G1 monoclonal antibody targeting VEGFR-2 32, and it has been confirmed as a novel second-line treatment for gastric adenocarcinoma and non-small cell lung adenocarcinoma 33, 34. In BTC, a clinical study of ramucirumab combined with pembrolizumab involving 26 samples suggested that although there is no significant survival improvement, the OS of PD-L1-positive patients is 11.3 months, which lays the foundation for the next study on how to develop immunotherapy regimens from the Endothelial cell direction 35. Existing research has suggested that TCF21 is capable of inhibiting tumor-associated angiogenesis via PI3K/Akt and ERK1/2 signaling pathways 36. Yokota et al. suggested that GPC1 (glypican-1) is significantly related to the clinical prognosis of CCA patients, and it is significantly expressed in CCA cells and vascular endothelial cells in CCA tissue. Thus, targeting GPC1 by ADC (Antibody-drug conjugates) can effectively inhibit the development of CCA. Grow 37. CD105 is highly expressed in vascular endothelial cells of pCCA, and it is an independent poor prognostic factor, which can serve as a feasible target for clinical treatment 38. Table 1 lists the role played by endothelial cells in the TME of CCA.

## Advances of Immune cells in CCA

The immune environment in the TME comprises different immune cell types, and different immune cells play different roles 39. To be specific, effector cells perform cell killing function, both in innate and adaptive immune responses. Antitumor cells in the adaptive immune response consist of CD4 ^+^ and CD8^+^ Teff cells, killing cancer cells by different mechanisms. There are also immunosuppressive cell populations in the TME (e.g., CD4^+^FOXP3^+^Tregs, myeloid-suppressor cells (MDSCs), macrophages, as well as some B cells). Antigen-presenting cells (e.g., intratumoral dendritic cells (DCs)) play a vital role in maintaining adaptive immune responses in the TME. The role played by immune cells in the tumor microenvironment can be either inhibition of tumor formation (antitumor microenvironment) or promotion of tumorigenesis (immunosuppressive microenvironment), which is dependent on contexts and tumor types.

### Adaptive immune response

Adaptive immune response is the whole process of autoactivation, proliferation and differentiation of antigen-specific T/B lymphocytes into effector cells after antigen stimulation *in vivo*, producing a series of biological effects.

#### CD8^+^ T cells in CCA

CD8^+^ T cells are expressed in 30%-35% T cells, recognize antigenic peptides composed of 8-10 amino acid residues, differentiate into cytotoxic T lymphocytes (CTLs) after activation, and have cytotoxic effects. They regulate the killing response of tumor cells by releasing perforin, granzyme and FAS/FASL transmembrane glycoprotein, trigger target cell apoptosis, and specifically kill target cells. In the TME, CTLs play an important role in the development of tumors, and the risk profile based on CD8^+^ T cell status is significantly related to CCA tumor status. It has been demonstrated that tumor-associated neutrophils are negatively related to CD8^+^ T cells, while low CD8 ^+^ T cells are significantly related to low OS 40. Wu et al. suggested that isocitrate dehydrogenase 1 mutations (mIDH1) promotes TET2 dependent induction of interferon γ (IFN-γ)-responsive genes in tumor cells by inhibiting the recruitment of stimulated CD8^+^ T cells and the expression of IFN-γ. CD8^+^ T cell depletion or tumor cell-specific ablation of IFN-γ receptor 1 thus causes improved tumor resistance. However, immune checkpoint activation limits the efficacy of mIDH1 inhibitors and restores the normal recruitment of CD8^+^ T cells thereby reducing tumor drug resistance and improving therapeutic efficacy 41. At the same time, the potential of CD8^+^ T lymphocytes as a prognostic marker for CCA has also been pointed out, and a significant increase in CD8^+^ T cell density, in the lymphoepithelial subtype of Epstein-Barr virus-associated CCA, is significantly related to a favorable outcome in ICC 42. Asahi et al. suggested that the number of CD8^+^ T cells in the outer edge of the tumor increased and then the number of HLA class I increased, and the role played by HLA class I expression was to present tumor antigen-derived peptides to the immune system, ultimately stimulating CD8^+^ T cells to show anti-tumor effects and optimize the prognosis of ICC 43.

Numerous studies on immune mechanisms of action and prognostic factors have paved the way for the use of CD8^+^ T cells for CCA treatment. Existing research has suggested the positive relationships between PD-L1 (programmed cell death ligand 1) expression, CD8^+^ tumor-infiltrating lymphocytes (TILs) infiltration, T cell inflammatory gene feature expression and estimated CD8 ^+^ TIL abundance in ICC tumor cells, while the characteristic genes of Wnt/β-catenin and TGF-β signaling pathways are enriched in tumors with a low proportion of CD69^+^ CD103^+^ tissue-resident memory-like CD8^+^ TIL. The above results reveal that the proliferation and recovery potential of CD69^+^ CD103^+^ double-positive, coupled with the expression of checkpoint receptors, can induce CD8 ^+^ T cell anti-tumor response and play a certain role in the treatment of ICC 44. B7-H4, or PD-L1, is a negative regulator of T cell responses, and it is expressed in different human tumors (e.g., breast and gastric cancers) 45, 46. Existing research has suggested that B7-H4 in CCA may play a negative regulatory role in T cells by inhibiting the recruitment or survival of lymphocytes in the TME, thus reducing the total number of infiltrating lymphocytes in the tumor stroma, especially CD8^+^ TILs. When the expression of B7-H4 is inhibited by the lentiviral vector encoding shRNA, the decrease of B7-H4 in tumor cells can be directly observed, and stable tumor cell lines with low expression of B7-H4 can be screened, and CD8 ^+^ T cell-mediated cytotoxicity is increased, providing a new idea for the treatment of CCA 47. Table 2 summarizes the current research on CD8^+^ T cells in CCA and confirms the importance of CD8^+^ T cells in the immune mechanism, prognostic factors and anti-CCA treatment of CCA.

#### CD4^+^ T cells in CCA

CD4^+^ T cells are a collective term for a class of mature T cells expressing CD4, which are suggested in 60% of T cells and some NKT cells, recognize CD4 antigenic peptides composed of 13- 17 amino acid residues, and after activation, differentiate into Th cells, while some CD4^+^ effector T cells have cytotoxic and immunosuppressive effects. CD4^+^ T cell infiltration was present in the interface area around the tumor in the liver surrounding the CCA tumor tissue, while CD8^+^ T cell infiltration was present inside the tumor. This finding was consistent with HCC T cell infiltration. This is independent of their histogenetic origin, but they are two distinct immune functions, suggesting that even lymphocytes of the same origin may have different functions in these tumor areas 48. Existing research has suggested that in patients with advanced local CCA, the infiltration of immune cells comprising CD4^+^ lymphocytes and other immune cells significantly increases compared with metastatic lesions. The above change in TME infiltration is primarily related to microvascular density (MVD) proliferation, and this proliferation is negatively related to CD4^+^ T cells 49. Similarly, Kida et al. analyzed peripheral blood samples from 41 patients with CCA and 10 healthy volunteers through flow cytometry. They suggested that t cells and immune checkpoint markers are enriched at the tumor margin compared with the tumor center, whereas a higher frequency of CD4^+^ T cells is an essential factor for the recurrence of CCA 50. It is necessary to conduct in-depth research on the relationship between CD4^+^T cells accumulation at the tumor margin of CCA and the prognosis. T cells and immune checkpoint markers are enriched at the tumor margin in comparison with the tumor center, and Carapeto et al. suggested that high PD-1 or CD4-inducible T cell costimulatory factors in lymphocyte-activated tumor centers contribute to poor survival in patients with ICC 51. A study on hepatobiliary tumors treated with hypofractionated proton therapy (HPT) radioactivity has suggested that overall survival (OS) is significantly related to CD4^+^ CD25^+^ T cell and CD4^+^ CD127^+^ T cell fractions in intrahepatic ICC, which reveals that CD4^+^ T cell and its related cytokines may be vital factors for the prognosis of CCA 52.

The above evidence suggests that the human body will develop a mutation-specific T lymphocyte response to CCA derived from epithelial cells. Tran et al. suggested that TILs from patients with metastatic CCA contained CD4^+^ T helper 1 (Th1) cells using whole exogenomic sequencing and were able to identify cancer-expressed erbb2-interacting protein (ERBB2IP) mutations. They transplanted patients with TILs containing about 25% mutation-specific multifunctional Th1 cells and experienced shrinkage of tumor lesions and higher disease stability. At the time of deterioration, the patient was treated with a pure population of > 95% mutation-responsive Th1 cells and again experienced regression of the tumor tissue. The above results suggest that the response of CD4 ^+^ T cells to mutant antigens can be used to mediate the regression of metastatic epithelial cell cancer tissues and may be a potential targeted therapy for CCA 53. Treatment with trametinib alone leads to the up-regulation of major histocompatibility complex (MHC-I) and PD-L1 on tumor cells *in vitro*, and it has been suggested that the combination of trametinib with anti-PD-1 drugs can lead to enhanced cytotoxicity of hepatic effector memory CD4 ^+^ T cells, reduced tumor burden, and improved survival in tumor-bearing mice 54. Table 2 lists more studies on CD4 ^+^ T lymphocytes. As depicted in this table, CD4^+^ T lymphocytes take on a great significance in the TME of CCA, and targeting these cells may achieve the purpose of immunotherapy.

#### Tregs in CCA

**Figure 4:**
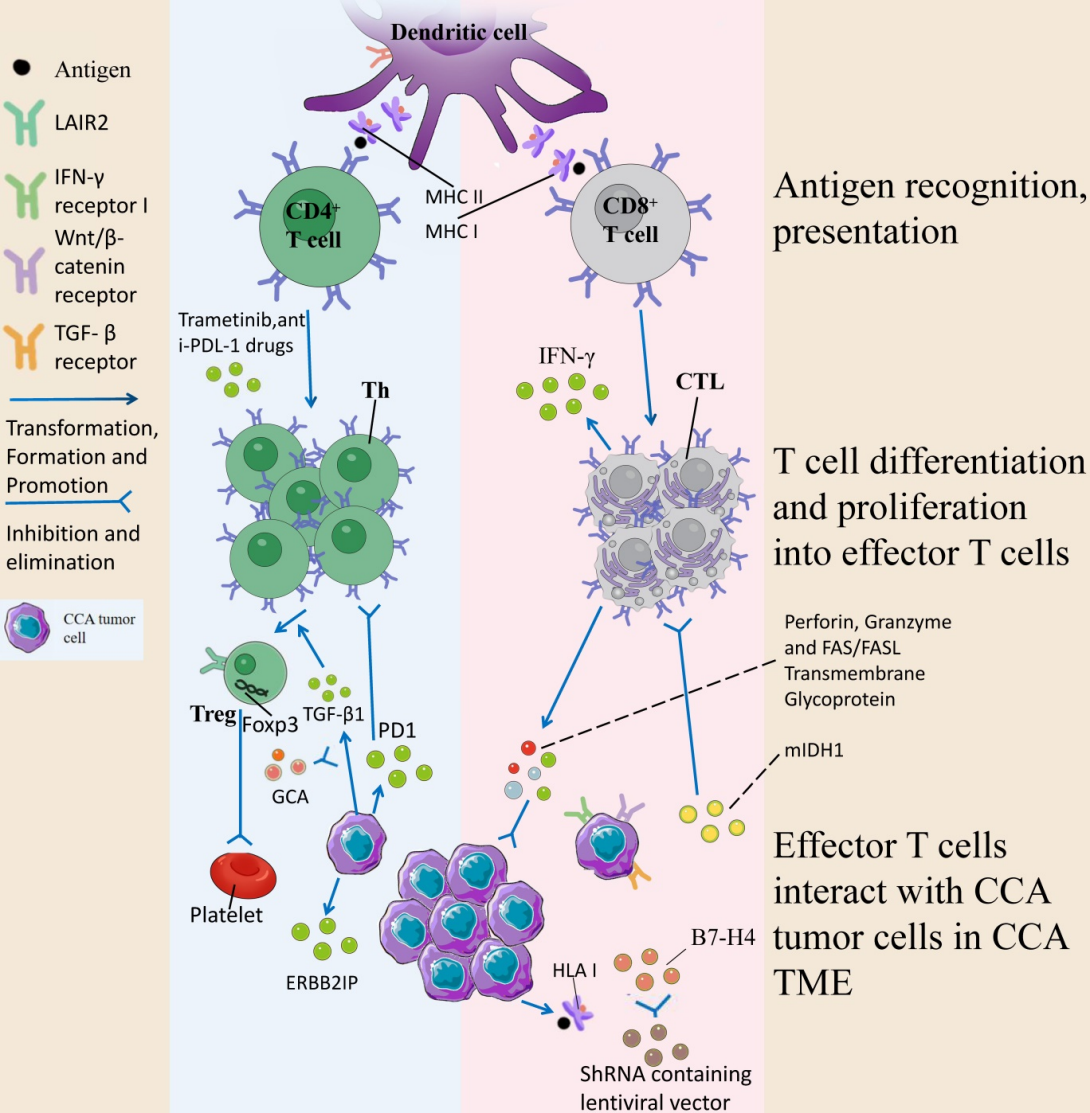
DCs activate naive T cells by presenting antigens by phagocytosis, initiate immune responses, secrete chemotactic cytokines, chemotactic T/B cells, and present antigens to CD8 ^+^ T cells/CD4 ^+^ T cells by MHC class I/II cells. Upon activation of CD8 ^+^ T cells, perforin is released, and granzymes and FAS/FASL transmembrane glycoproteins kill tumors. mlDH1 inhibited the recruitment of CD8 ^+^ T cells and the expression of IFN-γ, whereas mlDH1 inhibitor could contact this inhibition. At the same time, HLA class I is expressed and presents tumor antigen-derived peptides to the immune system, which ultimately stimulate CD8 ^+^ T cells to show anti-tumor effects. Inhibition of B7-H4 by lentiviral transcription encoding shRNA could enhance CD8 ^+^ T cell-mediated cytotoxicity. The response of CD4 ^+^ T cells to mutated ERBB2IP antigen can be used to mediate the degeneration of metastatic epithelial cell carcinoma tissues, trametinib can lead to the up-regulation of MHC-I and PD-L1 on tumor cells *in vitro*, and the combination of trametinib with anti-PD-L1 drugs can enhance the anti-tumor toxicity of hepatic effector memory CD4 ^+^ T cells. The increased expression of TGF-β1 in tumor cells induces Tregs heterogeneity in TME, forms an environment conducive to tumor cell proliferation, anti-apoptosis and angiogenesis, and promotes tumor progression, a mechanism that can be inhibited by the combination of GCA. LAIR2 expressed by Tregs blocks the binding of LAIR1 by competing ligands, interferes with platelet activation and adhesion, and inhibits the classical pathway of the complement system and the lectin pathway to kill pathogens.

Regulatory T cells (Treg) are usually CD4^+^CD25^+^FOXP3^+^T cells, accounting for 5% -10% of the total number of peripheral blood CD4 ^+^ T cells, and have a special mechanism of action in the TME of CCA, so we discuss them separately. FOXP3 is a transcription factor, FOXP3 deficiency can lead to severe autoimmune diseases 55, and Tregs are divided into natural regulatory T cells (nTregs) that come from thymic differentiation and peripherally derived inducible regulatory T cells (iTregs) according to the source 56. Tregs play an immunosuppressive role by down-regulating CD80 and CD86 on dendritic cells mainly by acting on CTLA-4 to inhibit effector cell activation 57. At present, there are relatively few studies on the role played by Tregs in CCA, but its important role in CCA has been confirmed: Kim et al. showed that the number of FOXP3 ^+^ CD4 ^+^ regulatory T cells in extrahepatic CCA was more than that in CCA, and the number of FOXP3 ^+^ CD4 ^+^ regulatory T cells at the tumor margin predicted a better prognosis 58. Kinoshita et al. suggested that Tregs accumulate in the tumor center of CCA, related to the expression of TGF-β1 in tumor cells. TGF-β1, a cytokine produced by tumor cells, induces Tregs heterogeneity in the TME, shapes an environment conducive to proliferation, anti-apoptosis and angiogenesis for tumor cells, makes tumor cells immune escape and promotes the development of CCA, while GCA (e.g., gemcitabine, and albumin-bound paclitaxel neoadjuvant therapy) can prevent the mechanism 59. Another study suggested that Treg-expressed leukocyte-associated immunoglobulin-like receptor 2 (LAIR2) can interfere with platelet activation and adhesion by blocking the binding of LAIR1 by competing ligands, inhibit the classical pathway and lectin pathway of the complement system to kill pathogens, and act as a clinical biomarker for evaluation of patients before immunotherapy, thereby predicting patient survival and tumor immune infiltration 60. The role played by Tregs in CCA is listed in Table 2. The interaction mechanism of T cells with CCA tumor cells in TME is detailed in Fig. 4.

#### B cells in CCA

TME is a complex structure composed of tumor cells, stromal and endothelial cells, and immune cells with dynamic interactions between the above cells. B cells are the second adaptive immune cell population suggested in the TME. B cells have been considered to have oncogenic properties for the past two decades 61. In renal cancer and glioblastoma, B cells have been demonstrated to be related to poor prognosis 62. It has also been shown that B cells play a role in TME by inhibiting tumors, such as B cells and CD4 ^+^ T follicular helper cells collaborating to promote anti-tumor CD8 ^+^ T cell responses^63^. Both of the above effects have a relatively clear basis and have different factual bases, and different infiltration patterns or TME induce the differentiation of B cells into different directions that can affect their final overall effects. In CCA TME, DNA hypermethylation of the EBF1 (early B cell factor 1) promoter inhibits EBF1 expression in CCA and induces CCA progression. Meanwhile, long-term oxidative stress would inhibit EBF1 expression in cholangiocytes as an adaptive response for cells to survive under continuous stress conditions, which in turn promotes CCA development^64^. However, there are also some studies with different findings: high B cells infiltration levels may be related to better OS and can serve as a biomarker for the evaluation of immune infiltration in CCA^60^, this happens to prove that B cells enhance T cell responses by producing antibodies, stimulatory cytokines, and chemokines, act as local antigen-presenting cells, and organize the formation of TLS that maintain immune responses, and TLS has been shown to be related to a positive prognosis in a variety of tumors^51^. Existing studies have illustrated the complex and heterogeneous role played by B cells in TME. Accordingly, further characterization of how B cell responses are initiated and regulated within the TME will help to develop clinical strategies for cancer immunotherapy. Contents regarding B cells are summarized in Table 2.

### Innate immune response

Innate immunity is an innate immune defense function formed by the body during germline development and evolution, i.e., non-specific defense function that has been possessed since birth, also known as non-specific immunity. It is a series of defense mechanisms formed by organisms during long-term evolution. Innate immunity is the physiological rejection of the body to a variety of antigenic substances.

#### DCs in CCA

As professional antigen-presenting cells, dendritic cells (DCs) contribute to the activation of T cells and are the initiators of the induction of T cell immune responses. Activation of the CD40/CD40L immune checkpoint leads to DCs maturation and affects the release of cytokines and chemokines 65. CD40 molecules are highly expressed on the surface of DCs. Existing research has suggested that CD40 agonist combined with anti-PD1 antibody treatment can significantly increase the number of DC cells in ICC tissue and limit tumor growth 66. Through flow cytometry analysis, Martín-Sierra et al. suggested that the number of Myeloid Dendritic Cells in the peripheral blood of CCA patients was significantly decreased, and the expression of TNF-α was decreased 67. In view of the important role played by DCs in inducing T cell immune responses, related studies have used DCs as a starting point to explore ways to enhance tumor immunity. Jiraviriyakul et al. suggested that DC cells sensitized with cell lysates produced by CCA cells after Honokiol treatment enhanced the antitumor immune response of T cells 68. In the tumor microenvironment, immunosuppressive cytokines (IL-10 and TGF-β) secreted by CCA cells affect DCs function and inhibit the activation of effector T cells 69. A clinical trial showed that DCs vaccine combined with activated T cell transfer can improve the prognosis of patients with CCA 70. Summary of the role played by DCs in the TME of CCA is listed in Table 3.

#### NK cells in CCA

Natural killer (NK) cells are a type of cytotoxic lymphocytes of the innate immune system. The markers commonly used to detect human NK cells consist of CD16, CD56, CD57, etc. Activated NK cells are capable of mediating direct cytotoxicity to tumor cells through “self-deletion” and ADCC effects, and they can perform immune monitoring functions by releasing cytokines (e.g., IFN-γ and TNF-α) 71. In patients with CCA, the immune monitoring function of NK cells is often affected, and this abnormality may be related to the alteration of KIR (killer cell immunoglobulin-like receptor) and HLA gene loci in patients with CCA 72. IL-12, IL-15, IFN-α/β, CXCL9/10 secreted by DCs play a vital role in the activation and recruitment of NK cells 73. CXCL9 produced by DCs, macrophages, endothelial cells, and others belongs to the ELR motif-negative CXC chemokine family. In the TME, endogenous CXCL9 is capable of regulating the number of tumor-infiltrating NK cells, and it is significantly related to the prognosis of patients with ICC 74. CCA is involved in regulating immune cells in the tumor microenvironment. Existing research has suggested that CCA cells can induce the apoptosis of CD4^+^, CD8^+^ T cells and CD56^+^ NK cells via the Fas/FasL pathway to produce immune evasion 75. To study how to maintain or increase the antitumor activity of NK cells, Hung et al. constructed a thioacetamide (TAA)-induced rat ICC model and suggested that the anti-Globo H antibody VK9 can facilitate the activation of NK cells in the tumor microenvironment, increase the Direct cytotoxicity and enhance ADCC function 76. Panwong et al. have suggested that cordycepin can increase the anti-CCA activity of NK-92, and TRAIL signaling may play a role in the immune regulation process of cordycepin 77. There has not been any immune cell therapy for CCA clinically, whereas studies in nude mice have confirmed the antitumor effect of NK cells on CCA cells, which provides a reference for the clinical application of NK cells therapy 78. Table 3 lists the role played by NK cells in the TME of CCA.

#### TANs in CCA

**Figure 5:**
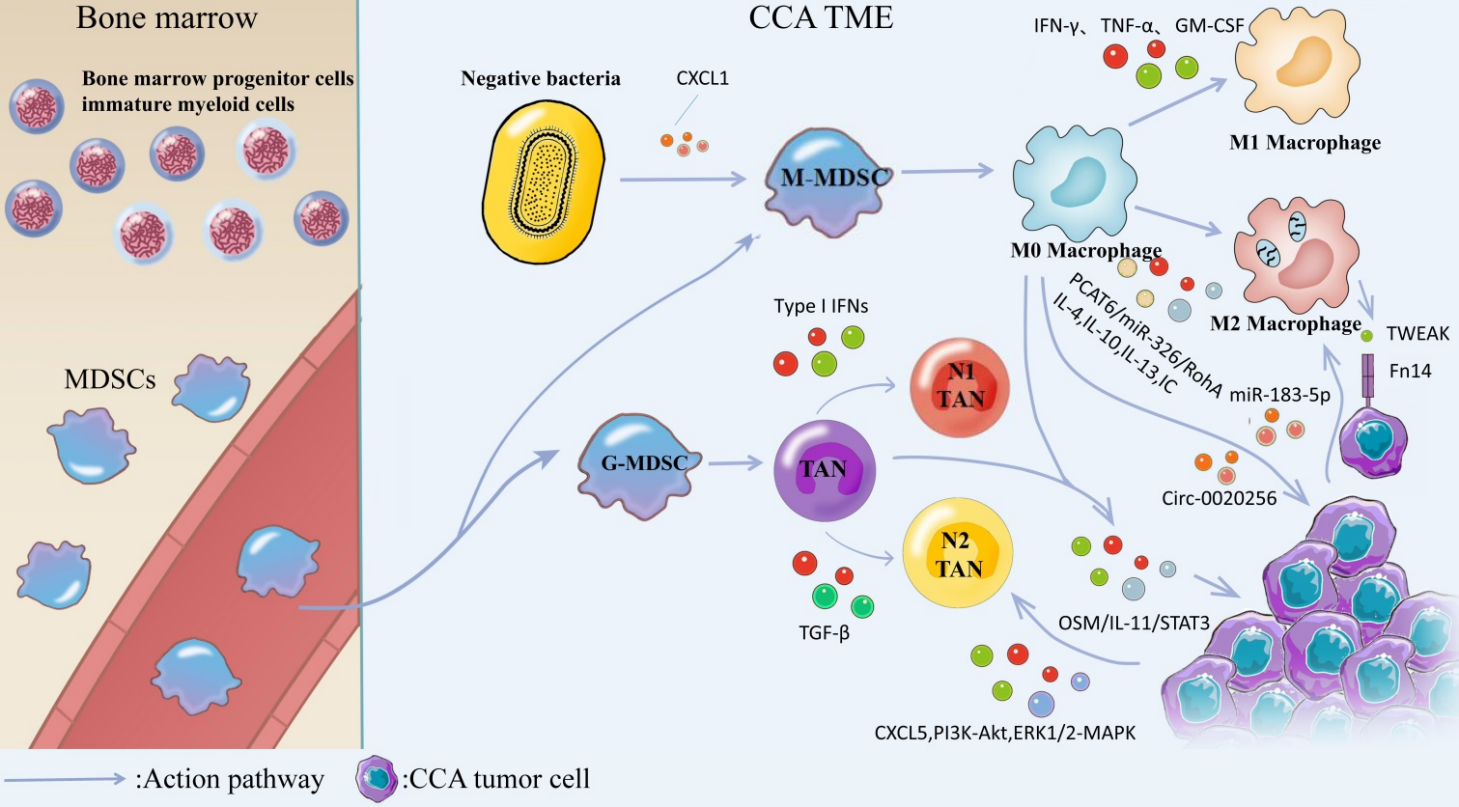
MDSCs are generated from bone marrow progenitor cellsimmature myeloid cells in the bone marrow and subsequently differentiate from blood vessels into M-MDSCs and G-MDSCs. Negative bacteria secrete CXCL1 to promote M-MDSCs transformation and generation, and M-MDSCs are converted into M0 Macrophages. Stimulation with cytokines such as IFN-γ, TNF-α, GM-CSF, or bacterial endotoxin induced the conversion of M0 Macrophages into M1 Macrophages, and IL-4, IL-10, IL-13, and IC induced the conversion of M0 Macrophages into M2 Macrophages. M2 macrophages induce the production of EMT through IL-10/STAT3 and AKT3/PRAS40 pathways. TWEAK produced by macrophages interacts with the Fn14 receptor on the surface of CCA cells and affects CCA progression. Tumor-derived exosome miR-183-5p up-regulates macrophage PD-L1 expression in TME through 315 miR-183-5p/PTEN/AKT/PD-L1 pathway, which promotes the development of immunosuppression in ICC. TAMs can interact with TANs, activate OSM/IL-11/STAT3 signaling pathway, and promote ICC progression. G-MDSCs were converted to N0 TANs and subsequently IFNs induced TANs to exhibit an anti-tumor N1 phenotype, whereas TGF-β could modulate TANs to exhibit a pre-malignant N2 phenotype. CXCL5 is highly expressed in CCA cells and promotes CCA progression by inducing neutrophil recruitment in tumor tissues via PI3K-Akt and ERK1/2-MAPK.

As professional phagocytes, neutrophils mainly mediate inflammatory responses, and their role in cancer has not been fully investigated. Neutrophils are affected by a variety of factors in the tumor microenvironment and have certain immunomodulatory functions 79. There are still many controversies about whether TANs play a tumor-promoting or tumor-suppressing role in cancer. Similar to the M1/M2 polarization of macrophages, TANs also have both N1 and N2 phenotypes when stimulated by different environmental factors. Type I IFNs induce TANs to display an anti-tumor N1 phenotype, while TGF-β can modulate TANs to a pro-cancer N2 phenotype 80. Neutrophils recruited by Methotrexate-loaded tumor-cell-derived microvesicles (MTX-TMP) perfusion display an N1 phenotype with the ability to kill extrahepatic CCA (eCCA) 81. CXCL5, as a pro-angiogenic CXC-type chemokine, is highly expressed in CCA cells, and induces the recruitment of neutrophils in tumor tissues through PI3K-Akt and ERK1/2-MAPK, promoting ICC progression 82. Gu et al. suggested that the infiltration of IL^+^17 cells and neutrophils in ICC tissue was closely related to tumor aggressiveness and increased risk of postoperative mortality in patients 83. By analyzing the postoperative specimens of 254 patients with CCA by immunohistochemical staining, Mao et al. suggested that the distribution of neutrophils in CCA tissue may indicate poor prognosis and affect the overall survival (OS) of patients 84. Research on TANs in the CCA microenvironment is limited, and more studies are needed to support their role in tumor immune regulation. Studies on the role played by TANs in the TME of CCA are listed in Table 4. The role played by two immune-related cells with dual effects on tumors in CCA TME is shown in Fig. 5.

#### Macrophages in CCA

Macrophages can generate canonical M1 activation and alternative M2 activation under different environmental stimuli. M1 macrophages are mainly induced by the stimulation of cytokines such as IFN-γ, TNF-α, GM-CSF or bacterial endotoxin, while IL-4, IL-10, IL-13, immune complexes (IC) and other factors trigger macrophage transformation to M2 phenotype 85. M1 macrophages secrete high levels of pro-inflammatory cytokines, produce highly reactive nitrogen and oxygen intermediates, and have strong tumoricidal activity. M2 macrophages are considered to be of great significance in facilitating tissue remodeling and tumor progression, and they have immunomodulatory functions 86. Macrophages accumulating within tumor tissue are termed tumor-associated macrophages (TAMs). Macrophages in the CCA immune microenvironment are the predominant infiltrating inflammatory cells, often exhibiting a M2 phenotype 87. PCAT6, as a LncRNA, is highly expressed in CCA patients, and previous studies have suggested that PCAT6/miR-326/RohA is an essential pathway that plays a role in M2 polarization of CCA-related macrophages 88. Th2 cytokine IL-4 also plays a role in stimulating the conversion of macrophages to the M2 phenotype 89. TAM-mediated inflammatory responses take on a critical significance in tumor transformation and progression, and they have been widely studied 90. A bioinformatics analysis based on the gene expression omnibus database suggested that activated dendritic cells, M2 macrophages and regulatory T cells (Tregs) were expressed at higher levels in ICC than normal tissues, while M1 macrophages, monocytes, and helper T cells were expressed at lower levels in ICC than in normal tissues 91. Thus, M2-TAMs has been the most studied macrophage cell type in the CCA tumor microenvironment. There is a close relationship between M2 TAMs and the development, immunosuppression, and clinical prognosis of ICC. M2 polarized macrophages play a role in promoting the growth and invasion of CCA and this potential mechanism may be the production of EMT (epithelial-mesenchymal transition) induced by M2 macrophages through the IL-10/STAT3 and AKT3/PRAS40 pathways 92, 93. Tumor necrosis factor-like weak inducer of apoptosis (TWEAK) produced by macrophages interacts with Fn14 receptors on the surface of CCA cells to influence CCA development 94. Macrophage-derived Wnt3a appears to play a role in the transformation of ICC cells 95. Several studies have shown that exosomes are important mediators linking TAMs and CCA. Circ_0020256 in TAM-derived exosomes can promote the proliferation, invasion, and migration of CCA 96. However, tumor-derived exosomal miR-183-5p upregulates the expression of macrophage PDL-1 in TME through the miR-183-5p/PTEN/AKT/PD-L1 pathway, thereby promoting the occurrence of immunosuppression in ICC 97. Hou et al. suggested that TAMs can interact with tumor-associated neutrophils (TANs) to activate the OSM/IL-11/STAT3 signaling pathway to promote ICC progression 98. Overall, TAMs mainly plays a role in promoting cancer in CCA. In order to inhibit the tumor-promoting effect of M2 macrophages, Ruffolo et al. built an orthotopic tumor-bearing mouse model of ICC and confirmed that anti-GM-CSF monoclonal antibody treatment can reverse tumor-induced M2 polarization of TAMs, thus stimulating the immune activity of T cells and preventing the establishment of an immunosuppressive microenvironment 99. TAMs are meaningful markers in evaluating the clinical prognosis of patients with ICC 100. Table 5 lists the existing studies on the role played by macrophages in the TME of CCA.

#### MDSCs

Myeloid-derived Suppressor Cells (MDSCs) are a heterogeneous group of bone marrow-derived cells that is a highly diverse cell population. MDSCs are classified into two categories, including mononuclear myeloid cells (M-MDSCs) (e.g., macrophages and dendritic cells (DCs)), as well as monocytes, and granulocytic myeloid cells (G-MDSCs) (e.g., terminally differentiated polymorphonuclear neutrophils, eosinophils, basophils, and mast cells 101. The major functions of MDSCs consist of immunosuppression, promoting tumor progression, upregulating tumor proliferation pathways, promoting angiogenesis, and supporting tumor spillage by disrupting the ECM 102. Extensive studies that have suggested a promoting effect of MDSCs on CCA, and the goal is to study inhibition of MDSCs to treat CCA. Previous findings have suggested that blocking TAMs and PD-L1 in CCA can reduce CCA progression, but G-MDSCs disable TAM and PD-L1 blockade by impairing T-cell-mediated immune escape responses. Loeuillard et al. suggested that dual inhibition of TAMs and G-MDSCs enhances immune checkpoint blockade (ICB), which highlights the therapeutic potential of ICB coupled to immunotherapy targeting immunosuppressive myeloid cells in CCA 103. *In vivo*, lipopolysaccharide (LPS) induces CXCR2^+^ and polymorphonuclear myeloid-derived suppressor cells (PMN-MDSC) accumulation through the production of TLR4-dependent CXCL1, controls CCA tumor cells to form an immunosuppressive microenvironment, and facilitates CCA tumor growth 104. Table 5 lists the studies on the role played by MDSC in the TME of CCA.

#### Mast cells in CCA

Mast cells were initially discovered by Paul Ehrlich in 1878 104. They originate from bone marrow hematopoietic progenitor cells migrating to and maturing in the surrounding tissue when not fully differentiated 105. Mast cells function is primarily divided into two aspects: from a physiological perspective, mast cells are involved in tissue remodeling and neovascularization 106, while from a pathological perspective, it is not only an important factor in IgE-mediated allergic diseases, but also involved in non-IgE-mediated allergic reactions 107, 108. A clinical specimen analysis suggested that immune cell infiltration consisting of CD8^+^, CD4^+^ lymphocytes, macrophages, and mast cells was significantly increased in locally advanced CCA patients compared with metastatic lesions, and this change in TME infiltration was related to a significant increase in MVD due to mast cell involvement in angiogenesis in local CCA lesions 49. However, the use of sodium chromolybdate can inhibit CCA proliferation, angiogenesis, and the presence of mast cell markers by blocking the mast cell c-Kit/SCF pathway 109. SCF (the stem cell factor) is a c-Kit ligand, and it is also known as steel factor. The c-Kit/SCF pathway is a vital pathway to facilitate tumor proliferation and angiogenesis 110. This study suggests that targeting c-Kit/SCF interaction by sodium chromomolybdate may be a potential therapeutic option to inhibit tumorigenesis and progression. Table 5 lists the role played by mast cells in the TME of CCA.

## Current Status of Immunotherapy for CCA

The treatment methods for CCA primarily comprise surgical treatment, radiation therapy, and systemic therapy in accordance with the guidelines for liver cancer issued by the US NCCN in 2022. To be specific, surgical treatment consists of subtotal hepatectomy, wedge resection and resection of the liver for ICC 8, pancreaticoduodenectomy for dCCA 111, as well as extensive hepatectomy for pCCA 112. However, the prognosis of surgical treatment does not achieve the ideal goal due to the complexity of the anatomy of hepatobiliary tumors and the multiple extrahepatic lymph node metastases, while functional reconstruction after resection surgery is a major problem. Radiation therapy is generally considered a supplement to surgical treatment. For instance, postoperative EBRT using conventional 3D-CRT or intensity-modulated radiotherapy is an option for resection of extrahepatic CCA and gallbladder cancer 113. However, as a radiosensitive organ, the liver and its surrounding tissues toxicity and other side effects caused by radiation therapy cannot be ignored 114. The recommended regimen given by the NCCN for the treatment of resectable CCA system, which has attracted much attention, is 5-fluorouracil, capecitabine or gemcitabine in combination with a chemotherapeutic drug, such as oxaliplatin, of which capecitabine is the preferred regimen for adjuvant therapy. Existing research has shown signs of improved survival in the experimental group compared with no capecitabine, whereas these signs did not achieve statistical significance 115. In unresectable CCA, the preferred regimen is gemcitabine combined with cisplatin 9, and although the mPFS can reach 8.0 months, the mOS is only 11.7 months, which is far from the expectations of clinicians. Another factor affecting the therapeutic effect of chemotherapeutic drugs is microsatellite instability-high (MSI-H) caused by the presence of mismatch repair (MMR) gene 116. However, if CCA belongs to MSI-H/dMMR type, it often has many gene mutations, but is sensitive to PD-1/PD-L1 inhibitor treatment. At the same time, with the increasing use of immunotherapies such as immune checkpoint inhibitors Immune checkpoint inhibitors (ICIs) in other cancers, such as PD-L1 monoclonal antibody combined with VEGFR monoclonal antibody regimen has become the first-line systemic treatment regimen for advanced HCC 117, methods for hepatobiliary tumors have also entered our horizon.

**Figure 6:**
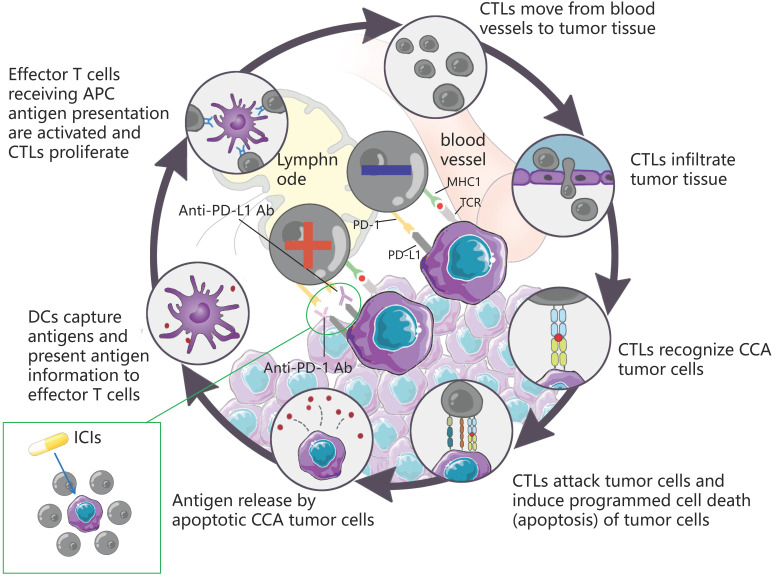
DCs capture antigens, present antigen information to effector T cells, and effector T cells receiving APC antigen presentation are activated and CTLs proliferate. CTLs move from blood vessels to tumor tissue, recognize tumor cells and infiltrate tumor tissue, attack tumor cells, induce programmed cell death (apoptosis) of tumor cells, and subsequently apoptotic tumor cells release antigens to form a dynamic cycle. PD-1 is an important immunosuppressive molecule. It prevents the immune system from killing cancer cells by regulating the immune system's response to human cells downward, as well as regulating the immune system and promoting self-tolerance by suppressing T-cell inflammatory activity. PD-L1 is a ligand expressed on the surface of tumor cells. PD-L1 is up-regulated in a variety of tumor cells. It binds to PD-1 on CTLs and inhibits CTLs proliferation and activation, so that CTLs are in an inactivated state and finally induce immune escape. ICIs can block the binding of PD-1 and PD-L1, up-regulate the growth and proliferation of CTLs, enhance the recognition of CTLs to tumor cells, activate their attack and killing function, and achieve anti-tumor effect by mobilizing the body 's own immune function.

ICI is capable of restoring T cell-mediated tumor cell killing and depleting regulatory Tregs by blocking immune checkpoint molecules (e.g., cytotoxic T lymphocyte antigen 4 (CTLA4), PD-L1, as well as PD-1. This mechanism of action has aroused wide attention of investigators. The understanding of the antitumor activity of PD-1/PD-L1 antibodies has been deepened over the past few years, and significant results have been achieved in the clinical treatment of HCC with similar biliary anatomy. In other words, camrelizumab combined with apatinib achieved ORR in 33.3% of cases of resectable HCC 118 in a recent phase II clinical trial. Accordingly, the application of immunotherapy in the treatment of CCA has attracted much attention. Durvalumab is an IgG1 κ monoclonal antibody, which acts as a PD-L1 blocker, can bind to PD-L1 on tumor cells, and block its interaction with PD-1 of T cells and antigen-presenting cells, thereby relieving PD-1/PD-L1-mediated immunouppression, promoting T cell proliferation, and strengthening the immune killing effect on tumors. A controlled trial involving 685 cases showed that Durvalumab combined with GEMCIS significantly prolonged OS compared with GEMCIS 119, and similar regimens were Camrelizumab combined with GEMOX and Pmbrolizumab combined with CAPOX 120, 121. The above studies showed that the combination of PD-L1 blocker and chemotherapeutic drugs had good efficacy in patients with advanced CCA. As an emerging treatment before the advent of immunotherapy, the combination of targeted therapy and immune drugs is also one of the hot directions for clinicians. In a study of lenvatinib in combination with PD-1 antagonists (including pembrolizumab, tislelizumab, sintilimab, camrelizumab, and toripalima) 14, the mOS and ORR reached 17.7 months and 42.1%, a result that is alarming and exciting. This may be related to the fact that targeted drugs act directly on the target thereby enhancing the therapeutic effect. In contrast, the therapeutic effect of ICIs alone, although good, is not as satisfactory as that of ICIs combined with chemical drugs or targeted drugs, such as nivolumab or pembrolizumab alone 122, 123, although the efficacy of ICIs alone is significant compared with traditional methods. However, not all the study results were positive. The mOS of ramucirumab combined with pembrolizumab was only 6.44 months, which did not show a significant improvement in survival rate, whereas the mOS of PD-L1 positive patients was 11.33 months 35, suggesting that PD-L1 positive can serve as a tumor marker to provide a reference for the designation of future immunotherapy methods. Table 6 lists more studies on immunotherapy in CCA. The mechanism of action of the ICIs is presented in Fig. 6.

Immunotherapy plays a certain role in the clinical treatment of CCA, whereas its efficacy and safety should be analyzed in more studies. Furthermore, the development of effective combination therapies with ICIs and chemotherapeutic drugs or targeted drugs may be the right direction to prolong the survival of CCA patients.

## Conclusion and perspectives

Novel treatments are urgently required due to the increasing incidence of CCA, limited efficacy of surgical treatment and chemotherapy, and poor prognosis. Targeted immunotherapy, a cutting-edge therapy in cancer treatment, is arousing rising attention. Although much remains to be explored, many studies have shown that immunotherapy has some advantages over conventional therapies, such as fewer side effects. In this review, some studies have proposed immunotherapeutic methods that are increasingly used in clinical practice (e.g., inhibiting MDSCs and enhancing immune checkpoint blocking). The involvement and regulation of cytokines and chemists, and the interaction between receptors and related ligands are of great significance in the function, maturation and differentiation of immune cells. The above factors, together with stromal cells and tumor cells, constitute TME. With the development of several technologies (e.g., single-cell RNA sequencing), the understanding of the complex mechanisms of TME should be deepened. Immunotherapy is not the end of cancer treatment. Instead, TME-based immunotherapy is the beginning of a new chapter in cancer treatment.

## Figures and Tables

**Table 1:** Summary of tumor stromal actions in the TME of CCA

Cells	The research direction	Result	Reference	Reference number
CAF	The immune mechanism	Hepatic stellate cell-derived CAFs are the main tumor-interacting population in ICC.	Affo et al. (2021)	17
CAF	The immune mechanism	Tumor exosomal miR-9-5p elicited IL-6 expression in vCAFs,thereby leading to epigenetic alterations in ICC.	Zhang et al. (2020)	18
CAF	Prognostic marker	Cellular senescence, represented by CAV1 levels, may be a marker of CAFs and a prognostic indicator of ICC through FOXP3^+^ TILs regulation. CAV1 expression in CAFs can be a therapeutic target for ICC.	Lan et al. (2021)	124
CAF	The immune mechanism	PDGF-D stimulates VEGF-C and VEGF-A production by fibroblasts, resulting in expansion of the lymphatic vasculature and tumor cell intravasation.	Cadamuro et al. (2019)	20
CAF	Prognostic marker	The ICC patients with immature CAF phenotype had a more aggressive feature and significantly poorer OS than those with mature phenotype.	Zhang et al. (2017)	125
CAF	The immune mechanism	Downregulation of tumor‐derived exosomal miR-34c induces cancer‐associated fibroblast activation to promote CCA progress.	Qin et al. (2021)	19
CAF	The immune mechanism	FAP Promotes Immunosuppression by Cancer-Associated Fibroblasts in the TME via STAT3-CCL2 Signaling.	Yang et al. (2016)	21
CAF	The immune mechanism	CAFs promoted proliferation, mi-gration, and invasion of CCA cells *in vitro* and boosted tumor growth *in vivo*. Furthermore, it was demonstrated that stroma enriched with α- SMA- positive CAFs was associated with poor prognosis of patients with ICC.	Sha et al. (2018)	126
CAF	Immunotherapy	The potential of repeated CAF-targeted PTT for the treatment of desmoplastic CCA after intra-tumoral administration.	Nicolás-Boluda et al. (2020)	127
CAF	The immune mechanism	IL-6 Secreted by CAFs Inhibits Autophagy and Reduces the Chemosensitivity of CCA Cells.	Thongchot et al. (2021)	128
CAF	The immune mechanism	ZEB1 plays a key role in CCA progression by regulating tumor cell-CAF crosstalk, leading to tumor dedifferentiation and CAF activation.	Lobe et al. (2021)	129
CAF	Prognostic marker	FAP overexpression is evident in dCCA. There was a positive association between epithelial FAP expression and better survival.	Byrling et al. (2020)	130
CAF	The immune mechanism	Treatment with LTB4 that were elevated in CAF-educated MDSCs or blockade of BLT2 that was preferentially expressed in stem-like ICC cells significantly reduced stemness-enhancing effects of CAF-educated MDSCs.	Lin et al. (2022)	131
CAF	The immune mechanism	To escape EGFR-TKI treatment, CCA tumor cells develop an adaptive mechanism by undergoing an IR/IGF1R-dependent phenotypic switch.	Vaquero et al. (2018)	132
CAF	immunotherapy	PlGF blockade leads to a reduction in intratumorous hypoxia and metastatic dissemination, enhanced chemotherapy sensitivity and increased survival in mice- bearing aggressive ICC.	Aoki et al. (2021)	133
CAF	Immunotherapy	Conditioned culture medium from CCA-derived CAFs further stimulated IL-6 secretion in CCA cells and promoted the migration of invasive cholangiocytes, while the nutrient resveratrol strongly counteracted this effect.	Thongchot et al. (2018)	134
CAF	Prognostic marker	High level of interleukin-33 in cancer cells and cancer-associated fibroblasts correlates with good prognosis and suppressed migration in CCA.	Yangngam et al. (2020)	22
CAF	The immune mechanism	Fibroblast growth factor receptor inhibition induces loss of matrix MCL1 and necrosis in CCA.	Kabashima et al. (2018)	135
CAF	The immune mechanism	CCA Cells Secreting PDGF-D Strongly Stimulate Fibroblast Recruitment, an Effect That Is Significantly Reduced by PDGFRb Antagonism and by PDGF-D siRNA.	Cadamuro et al. (2013)	136
CAF	Immunotherapy	MiR-206 suppresses the deterioration of intrahepatic CCA and promotes sensitivity to chemo-therapy by inhibiting interactions with stromal CAFs.	Yang et al. (2022)	24
CAF	The immune mechanism	The microRNA-15a-PAI-2 axis in CCA-associated fibroblasts promotes migration of cancer cells.	Utaijaratrasmi et al. (2018)	137
CAF	Immunotherapy	Nintedanib inhibited the cancer-promoting effect of CAFs via the suppression of CAF activation and secretion of cancer-promoting cytokines.	Yamanaka et al. (2020)	23
CAF	Immunotherapy	Navitoclax treatment triggered CAF apoptosis, diminishing expression of the desmoplastic extracellular matrix protein tenascin C, suppressing tumor outgrowth, and improving host survival.	Mertens et al. (2013)	138
Endothelial cell	immunotherapy	Anti-Glypican-1 Antibody-drug Conjugate targets CCA cells and GPC1 in vascular endothelial cells, and directly or indirectly inhibits CCA growth by inhibiting tumor angiogenesis.	Yokota et al. (2021)	37
Endothelial cell	Prognostic marker	CD105 is a tumor-associated endothelial cell marker. High expression of CD105 was independently associated with poor survival in patients with CCA.	Nair et al. (2020)	38
Endothelial cell	The immune mechanism	Plasmalemma vesicle-associated protein (PLVAP) is associated with angiogenesis in CCA. DKK1 secreted by CCA cells promotes tumor angiogenesis through the DKK1/CKAP4/PI3K/PLVAP pathway.	Wang et al. (2021)	28
Endothelial cell	The immune mechanism	ERK5 is highly expressed in human CCA cells, regulates the content of VEGF and angiopoietin 1 in the tumor microenvironment, and induces angiogenesis in tumor-associated endothelial cells.	Gentilini et al. (2021)	30
Endothelial cell	The immune mechanism	Circ-CCAC1 of CCA-derived EVs was transferred into CCA vascular endothelial monolayer cells, disrupting endothelial barrier integrity and inducing angiogenesis.	Xu et al. (2021)	31
Endothelial cell	The immune mechanism	Pcca cell-derived HMGB1 upregulates VEGFR2 expression in vascular endothelial cells and induces ectopic angiogenesis by *in vitro* and *in vivo* experiments.	Xu et al. (2019)	29
Endothelial cell	The immune mechanism	The secretion of PDGF-D by CCA cells resulted in increased levels of VEGF-C and VEGF-A secreted by fibroblasts and increased endothelial cell permeability.	Cadamuro et al. (2019)	20
Endothelial cell	The immune mechanism	TCF21 was decreased in CCA tissues or cell lines compared to normal tissues. TCF21 exerts anti-angiogenic activity through PI3K/Akt and ERK1/2 signaling pathways.	Duan et al. (2019)	36
Endothelial cell	The immune mechanism	THBS1, THBS2 and PEDF are up-regulated in the ICC microenvironment. The ability of THBS1, THBS2 and PEDF to inhibit endothelial cell angiogenesis was demonstrated by *in vivo* experiments.	Carpino et al. (2021)	139
Endothelial cell	The immune mechanism	eNOS is upregulated in CCA tissues and their cell lines, promoting angiogenesis and metastasis in CCA.	Suksawat et al. (2017)	26

**Table 2:** Summary of the role of CD8 ^+^ T Cells, CD4 ^+^ T Cells and Tregs in the TME of CCA

Cells	The research direction	Result	Reference	Reference number
CD8^+^ T Cell	Prognostic marker	The LEL subtype of EBV aICC, which had a significantly increased density of CD8^+^T cells, was significantly related to favorable outcome in ICC.	Huang et al. (2021)	42
CD8^+^ T Cell	Prognostic marker	Higher number of CD8^+^T cells in the outer boundary area, means lower number of HLA class I antigen value and predicts better prognosis in ICC patients.	Asahi et al. (2020)	43
CD8^+^T Cell	Prognostic marker	The density of CD8^+^ T-cells in the tumor and stroma correlated with OS and DFS. The number of CD8-positive TILs was significant independent risk factors for a poorer prognosis.	Deng et al. (2021)	140
CD8^+^T Cell	Prognostic marker	CD8^+^ TILs levels were significantly correlated with low LCR (lymphocyte-C-reactive protein ratio), while low LCR was significantly correlated with age, high crp-albumin ratio, and advanced disease, which is an indicator of postoperative prognosis in ICC patients.	Miyazaki et al. (2021)	141
CD8^+^T Cell	Prognostic marker	eCCA associated neutrophils were inversely correlated with CD8 ^+^ T cells.	Kitano et al. (2018)	40
CD8^+^T Cell	Prognostic marker	TILs are associated with prognosis of ICC patients after complete surgery. CD3^+^ and CD8^+^ infiltrate is associated with higher survival and lower recurrence risk.	Vigano et al. (2019)	142
CD8^+^T Cell	The immune mechanism	The expression of B7-H4 serves a role in shielding tumors from immune surveillance by suppression of tumor-infiltrating CD8^+^ T lymphocytes in CCA.	Zhao et al. (2016)	47
CD8^+^T Cell	The immune mechanism	The mIDH1 supports CCA tumor maintenance through suppressing CD8 ^+^ T cell activity, Immune checkpoint activation can inhibit the effect.	Wu et al. (2022)	41
CD8^+^T Cell	Immunotherapy	CD69^+^CD103^+^TRM-like CD8^+^TILs represent prominent tumour-specific immune responses and hold promise as a potential therapeutic target in ICC patients.	Kim et al. (2021)	44
CD4^+^T Cell	Immunotherapy	TILs from a patient with metastatic CCA contained CD4^+^ Th1 cells recognizing a mutation in ERBB2IP expressed by the cancer reduces target lesions and prolongs disease stability	Tran et al. (2014)	53
CD4^+^T Cell	Immunotherapy	The combination of Trametinib and PD-L1 can lead to enhanced cytotoxicity of hepatic effector memory CD4 ^+^ T cells, reduced tumor burden, and improved survival in tumor-bearing mice.	Wabitsch et al. (2021)	54
CD4^+^T Cell	The immune mechanism	In patients with advanced local CCA, the infiltration of CD4^+^ lymphocytes and CD8^+^ lymphocytes, macrophages, and mast cells was significantly increased compared with metastatic lesions, and this change in TME infiltration was mainly associated with MVD proliferation, and this proliferation was negatively correlated with CD8^+^ and CD4^+^ cells.	Tamma et al. (2019)	49
CD4^+^T Cell	The immune mechanism	T-cell and immune checkpoint markers are enriched at the tumor margins compared to the tumor center. high PD-1 or lymphocyte-activation gene 3 and low CD3/CD4/inducible T-cell costimulator specifically in the tumor center as associated with poor survival.	Carapeto et al. (2022)	51
CD4^+^T Cell	The immune mechanism	The percentage of CD4^+^CD45RO^+^ T cells producing IL-22 what significantly higher in liver fluke-infected patients than in healthy controls or even CCA patients without liver fluke infection. In samples was significantly higher in patients with CCA than in patients without it, and the percentages in these two groups were significantly higher than that in controls.	Su et al. (2017)	143
CD4^+^T Cell	The immune mechanism	CCAs contained a heterogeneous amount of TILs, composed mainly of CD3^+^ T cells with a predominance of CD8^+^ cells in the tumor tissue and of CD4^+^ cells in the interface region.	Kasper et al. (2009)	48
CD4^+^T Cell	Prognostic marker	T cells and immune checkpoint markers were enriched at the tumor margin compared to the tumor center. At the same time, a higher frequency of CD4 ^+^ T cells is an important factor in CCA recurrence.	Kida et al. (2021)	50
CD4^+^T Cell	Prognostic marker	Longer OS was significantly correlated with greater CD4^+^CD25^+^ T cell and CD4^+^CD127^+^ T cell fractions at baseline only in ICC patients.This findings suggest a differential relevance of immuno-modulation by HPT in these liver cancers.	Grassberger et al. (2018)	52
Treg	Prognostic marker	The density of FOXP3^+^ CD4^+^ regulatory T cells was higher in eCCA regardless of the tumor site is a key metric associated with clinical outcomes in BTC patients.	Kim et al. (2021)	58
Treg	Prognostic marker	LAIR2 is expressed by Tregs and some GZMB ^+^ CD8^+^ T cells, is associated with survival, and has increased expression in tumor tissues, which can be used as a prognostic marker for CCA.	Chen et al. (2021)	60
Treg	The immune mechanism	The mIHC demonstrated that both T follicular helper and regulatory T cells were significantly increased in intra-tumoral TLSs compared to peri-tumoral counterparts.	Ding et al. (2022)	144
Treg	The immune mechanism	The cells at the Biliary Cancer cancer Center secrete TGF-β1 and induce Tregs, which creates an immune-suppressive environment.	Kinoshita et al. (2020)	59
B Cell	The immune mechanism	Oxidative stress-mediated reduction in EBF1 expression induces CCA progression.	Armartmuntree et al. (2018)	64
B Cell	Prognostic marker	PNOC expressed by infiltrating B cells in CCA predicts better survival of patients.	Chen et al. (2021)	60

**Table 3:** Summary of the role of DCs and NK cells in the TME of CCA

Cells	The research direction	Result	Reference	Reference number
DC	Immunotherapy	CD40 mediates DC activation in ICC. Anti-CD40/PD-1 combination therapy significantly inhibited tumor growth in murine ICC models.	Diggs et al. (2021)	66
DC	Immunotherapy	The 5-year PFS and OS of ICC patients who received dendritic cell vaccine plus activated T cell transfer after surgery were longer than those of the control group. It shows that this therapy has the potential to become the standard adjuvant therapy for ICC.	Shimizu et al. (2012)	70
DC	Immunotherapy	Tumor lysates generated after Honokiol treatment of CCA cells can enhance the antigen presentation of DCs and stimulate the specific killing effects of T cells.	Jiraviriyakul et al. (2019)	68
DC	The immune mechanism	*In vitro* activation of CD40/CD40L immune checkpoints can affect the immune regulation function of DCs, providing a new entry point for the immunotherapy of malignant tumors such as CCA.	Sadeghlar et al. (2021)	145
DC	The immune mechanism	IL-10 and TGF-β secreted by CCA cells inhibit the function of DCs. Inhibition of IL-10 and TGF-β receptors on DCs by specific neutralizing antibodies can enhance the activity of effector T cells.	Thepmalee et al. (2018)	69
DC	Prognostic marker	The degree of infiltration of BDCA2^+^ plasmacytoid dendritic cells (PDCs) in peritumoral tissue may serve as a novel prognostic predictor in ICC patients undergoing radical resection.	Hu et al. (2020)	146
DC	The immune mechanism	Self-differentiated dendritic cells were transduced with short-hairpin RNAs lentiviral to knock down TGF-βRII and IL-10RA mRNAs, and the anti-CCA activity of T cells was enhanced.	Thepmalee et al. (2020)	147
NK	Immunotherapy	In a randomized placebo-controlled phase I clinical trial, oral administration of Atractylodes lancea (Thunb) DC. (AL) significantly increased the number of NK cells in blood samples from patients with CCA, showing a favorable anticancer effect.	Kulma et al. (2021)	148
NK	Immunotherapy	Globo H is highly expressed in ICC. The anti-Globo H mAbVK9 can increase the number of NK cells in the tumor microenvironment and limit tumor growth, indicating that Globo H is a valuable therapeutic marker for ICC.	Hung et al. (2022)	76
NK	Immunotherapy	Cordycepin can increase the expression of DR4 and DR5 in CCA cell line KKU-213A, thereby inducing NK cell cytotoxicity.	Panwong et al. (2021)	77
NK	The immune mechanism	*In vitro* studies showed that ICC cells induced apoptosis of T cells and NK cells through the Fas/FasL pathway.	Carnevale et al. (2017)	75
NK	Prognostic marker	The results of immunohistochemical analysis showed that the high expression of CXCL9 in ICC was significantly correlated with the infiltration of NK cells. CXCL9 can also serve as an effective prognostic marker.	Fukuda et al. (2020)	74
NK	The immune mechanism	NK cells in CCA express KIR receptors. Multiple alterations of KIR and HLA loci in patients with CCA may affect the immune surveillance function of NK cells.	Cornillet et al. (2019)	72
NK	Immunotherapy	*In vitro* and *in vivo* experiments have shown that NK cells have significant cytotoxicity to CCA cells, providing a research basis for the clinical application of NK cell therapy.	Jung et al. (2018)	78

**Table 4:** Summary of the role of macrophages and neutrophils in the TME of CCA

Cells	The research direction	Result	Reference	Reference number
Macrophages	The immune mechanism	The spatial distribution of TANs and TAMs correlated with each other in samples from patients with intrahepatic CCA. STAT3 is an important molecule in the interaction between TANs and TAMs to promote the progression of ICC.	Zhou et al. (2021)	98
Macrophages	Immunotherapy	Periostin secreted by intrahepatic CCA stem cells (ICSCs) promotes the recruitment of TAMs in the TME, providing a new target for immunotherapy.	Zeng et al. (2018)	149
Macrophages	The immune mechanism	The presence of macrophages exacerbates the cytotoxicity, DNA damage and ROS generation of 1,2-Dichloropropane (1,2-DCP) on cholangiocytes, which may be the underlying mechanism of 1,2-DCP-induced carcinogenesis of CCAs.	Ekuban et al. (2021)	150
Macrophages	The immune mechanism	M2-TAM may promote CCA metastasis through EMT process in the early stage of live Hodgella opioides (Ov)-induced CCA (CCA). High density of M2 TAMs in patient CCA tissue was significantly associated with extrahepatic metastasis.	Thanee et al. (2015)	92
Macrophages	The immune mechanism	M2 macrophage-derived TGFβ1 promotes CCA progression and chemoresistance through aPKCɩ-mediated NF-κB signaling pathway. CCL5 secreted by CCA cells undergoing aPKCɩ-induced EMT in turn regulates macrophage recruitment and polarization.	Yang et al. (2022)	151
Macrophages	The immune mechanism	The lncRNA PCAT6 (PCAT6) plays an important role in regulating the function and differentiation of immune cells. In a CCA xenograft mouse model, the PCAT6/miR-326/RohA pathway regulates M2 macrophage polarization.	Tu et al. (2020)	88
Macrophages	The immune mechanism	Interaction between M2 macrophages and ICC, M2 polarized macrophages induce EMT in CCA cells via IL-10/STAT3.	Yuan et al. (2020)	152
Macrophages	The immune mechanism	In the ICC mouse model, the recruitment of hepatic macrophage-derived Wnt3a due to inflammation may promote the malignant transformation of hepatocytes into ICC cells.	Saito et al. (2018)	95
Macrophages	Immunotherapy	The number of CD68^+^ TAMs infiltrated in tumor tissue was associated with OS in patients undergoing resection. Anti-GM-CSF therapy can relieve the establishment of an immunosuppressive microenvironment through the repolarization of TAMs and MDSCs.	Ruffolo et al. (2022)	99
Macrophages	The immune mechanism	CCA-sphere conditioned medium constructs an immune niche suitable for the growth of CCA by regulating macrophages.	Raggi et al. (2017)	153
Macrophages	The immune mechanism	Exosomes are important mediators of the crosstalk between tumor cells and the tumor microenvironment. miR-183-5p upregulates PD-L1 expression in macrophages through the miR-183-5p/PTEN/AKT/PD-L1 pathway, mediating CCA Immunosuppression in the process.	Luo et al. (2022)	97
Macrophages	The immune mechanism	The TAM-secreted exosome Circ_002056 regulates the proliferation, migration and invasion of CCA cells in the tumor microenvironment through the Circ_002056/miR-432-5p/E2F3 axis. CircRNAs produced by tumor-associated macrophages can serve as a new molecular target for clinical therapy.	Chen et al. (2022)	96
Macrophages	Prognostic marker	Immunohistochemical techniques were used to assess the abundance of TAMs in tumor samples from patients with intrahepatic CCA. The number of TAMs in the tumor invasive front is a meaningful prognostic marker in routine histopathological evaluation.	Atanasov et al. (2017)	100
Macrophages	Prognostic marker	In ICC patient tumor samples, the number of macrophages was positively correlated with the number of blood vessels and regulatory T cells, but not with the OS of the patients.	Hasita et al. (2010)	154
Macrophages	Prognostic marker	Intratumoral M2 macrophages are associated with OS in patients with CCA, suggesting the possibility of targeting macrophages for therapy.	Kunk et al. (2021)	155
Macrophages	The immune mechanism	When ICC cells were co-cultured with M2-TAMs, the core cytokines (GM-CSF, TNF-α, ICAM-1, IL-6) secreted by M2-TAMs activated the AKT3/PRAS40 signaling pathway to promote EMT in ICC cells.	Sun et al. (2020)	93
Macrophages	The immune mechanism	1,2-DCP stimulates macrophages to induce high expression of the pro-inflammatory factor TNF-α, thereby inducing CCA.	Zong et al. (2019)	156
Macrophages	The immune mechanism	Compared with normal liver tissue, Wnt mRNA was significantly elevated in CCA tissue. LPS induces upregulation of Wnt3 mRNA in macrophages and induces CCA through the Wnt/β-catenin pathway.	Loilome et al. (2014)	157
Neutrophils	The immune mechanism	Overexpression of CXCL5 in CCA recruits neutrophils to aggregate and promote ICC growth and metastasis.	Zhou et al. (2014)	82
Neutrophils	Prognostic marker	Analysis of different microanatomical regions of intrahepatic CCA by tissue microarray and immunohistochemistry demonstrated that neutrophils were an independent factor for evaluating the prognosis of patients with intrahepatic CCA.	Gu et al. (2012)	83
Neutrophils	Immunotherapy	Methotrexate-containing plasma membrane microvesicles induce neutrophil aggregation in eCCA and relieve obstructive eCCA.	Gao et al. (2020)	81
Neutrophils	Prognostic marker	CD15 neutrophil staining was performed on tissue sections by immunohistochemical staining. The level of neutrophils in CCA tissues was higher than that in adjacent tissues. The expression of CD15 was significantly associated with DFS and OS in patients with CCA.	Mao et al. (2015)	84

**Table 5:** Summary of MDSCs and Mast cells in the TME of CCA

Cells	The research direction	Result	Reference	Reference number
MDSC	The immune mechanism	G-MDSCs accelerate CCA progression by impairing T cell-mediated immune escape responses and disabling TAM and PD-L1 blockade.	Loeuillard et al. (2020)	102
MDSC	The immune mechanism	Conditioned media (CM) from CAF-educated MDSCs drastically promoted tumorsphere formation efficiency and stemness marker gene expression in ICC cells. CAF-CM stimulation increased expression and activity of 5-LO in MDSCs, while 5-LO inhibitor impaired the stemness-enhancing capacity of MDSCs *in vitro* and *in vivo*. Furthermore, IL-6 and IL-33 primarily expressed by CAFs mediated hyperactivated 5-LO metabolism in MDSCs.	Lin et al. (2022)	131
MDSC	The immune mechanism	*In vivo*, bacteria/(LPS) induce CXCR2 ^+^ PMN-MDSC accumulation through TLR4-dependent CXCL1 production, control CCA tumor cells to form an immunosuppressive microenvironment, and promote CCA tumor growth.	Zhang et al. (2020)	18
Mast cell	The immune mechanism	In locally advanced CCA patients, there is a significant increase of immune cell infiltrate constituted by CD8^+^ and CD4^+^ lymphocytes, macrophages and mast cells as compared to the metastatic ones.	Tamma et al. (2019)	49
Mast cell	The immune mechanism	Inhibition of mast cell-derived histamine decreases human CCA growth and differentiation via c-Kit/SCF-dependent signaling.	Johnson et al. (2016)	109
Mast cell	The immune mechanism	Mast cells increase during carcino- genesis in HCC and ICC, and they may play a role in fibrosis or tumor immunology in HCC and ICC.	Terada et al. (2000)	158

**Table 6:** Summary of Immunotherapy in CCA

Study	Immunotherapy regimen	Type of mechanism of action	Sample size	Median follow-up duration, months	Disease status	mPFS, months	mOS, months	ORR, %	Significantly improved survival compared to historical controls	Reference number
Arkenau et al. (2018) NCT02443324	Ramucirumab plus Pembrolizumab	IgG1 VEGFR-2Antagonists and IgG4 PD-1 Antagonists	26	15.7	Advanced BTC	1.64	6.44	3.8	No	35
Chen et al. (2020) NCT03486678	Camrelizumab plus GemOx	IgG4-κ; PD- 1 Antagonists and Chemotherapy	36	11.8	Advanced BTC	6.1	11.8	80.0 (patients with PD- L1 TPS ≥1%) 53.8 (patients with PD- L1 TPS <1%)	Yes	120
Chen et al. (2021) NCT03092895	Camrelizumab plus FolFox4	IgG4-κ; PD- 1; Antagonists and Chemotherapy	29	8.7	Advanced BTC	5.5	12.9	10.3	Yes	159
	Camrelizumab plus GemOx		63			3.8	13.6	19.0		
Zhang et al. (2021) ChiCTR2100044476	Lenvatinib plus PD-1 inhibitors (pembrolizumab/tislelizumab/sintilimab/camrelizumab/toripalimab)	Tyrosine kinase inhibitor and PD-1 Antagonists	38	13.7	Unresectable BTC	8.0	17.7	42.1	Yes	14
Oh et al. (2022) NCT03875235	Durvalumab plus GemCis	IgG1-κ; PD- 1 Antagonists and Chemotherapy	341	13.7	Advanced BTC	7.2	12.8	26.7	Yes	160
	Placebo plus GemCis	Chemotherapy	344	12.6		5.7	11.5	18.7		
Monge et al. (2022) NCT03111732	Pmbrolizumab plus CapOx	IgG4-κ; PD- 1 Antagonists and Chemotherapy	11	34.8	Advanced BTC	4.1	9.9	NA	Yes	121
Kim et al. (2020) NCT02829918	Nivolumab	IgG4; PD- 1 Antagonists	54	12.4	Advanced BTC	3.7	14.2	22.0	Yes	122
Piha-Paul et al. (2020); NCT02628067, NCT02054806	Pembrolizumab	IgG4; PD- 1 Antagonists	104	7.5	Advanced BTC	2.0	7.4	5.8	Yes	12
			24	5.7		1.8	5.7	13		
Lee et al. (2020) NA	Pembrolizumab	IgG4; PD- 1 Antagonists	51	3.8	Metastatic BTC	2.1	6.9	17	Yes	123
Goyal et al. (2020) NCT02375880	DKN-01 plus GemCis	A humanized monoclonal antibody targeting DKK1 and chemotherapy	51	NA	Advanced BTC	8.7	12.4	21.3	Yes	161
Feng et al. (2020) NCT03311789	Nivolumab plus GemCis	IgG4-κ; PD- 1 Antagonists and Chemotherapy	32	12.8	Unresectable or Metastatic BTC	6.1	8.5	33.3	Yes	13
Xie et al. (2019) NCT01853618	Tremelimumab plus Microwave ablation	CTLA-4 and Targeted therapy	20	NA	Refractory BTC	3.4	6.0	NA	Yes	162
Boilève et al. (2021) NCT03704480	Durvalumab plus Tremelimumab plus paclitaxel	IgG1-κ; PD- 1 Antagonists plus CTLA-4 Antagonists plus Chemotherapy	20	NA	Advanced BTC	NA	NA	NA	NO	163

## Abbreviations

CCA: cholangiocarcinoma
TME: tumor microenvironment
APC: annual percentage change
BTC: biliary tract cancer
PD-1: programmed cell death protein 1
PD-L1: programmed cell death-ligand 1
OS: overall survival
mOS: median overall survival
CAFs: cancer-associated fibroblasts
ECM: extracellular matrix
HSCs: hepatic stellate cells
MET: mesenchymal to epithelial transition factor
IL-6: interleukin-6
vCAF: vascular cancer-associated fibroblast
IL-6R: interleukin 6 receptor
miR: micro RNA
Wnt: wingless/integrated
VEGF: vascular endothlial growth factor
PDGF: platelet-derived growth factor
FAP: fibroblast activation protein
STAT: signal transducer and activator of transcription
CCL: c-c motif chemokine ligand 2
eNOS: endothelial nitric oxide lyase
PGE: prostaglandin E
TGF: transforming growth factor
DKK-1: dickkopf-related protein 1
VEGFR: vascular endothelial growth factor receptor
HMGB: high mobility group box
ERK: extracellular signal-regulated kinase
HUVEC: human umbilical veins
Circ-CCAC1: CCA-associated circRNA-1
LEC: lymphatic endothelial cells
TCF: transcription factor
GPC: phosphatidylinositol proteoglycan
ADC: antibody-drug conjugates
FOXP3: forkhead box P3
MDSCs: myeloid-suppressor cells
DCs: dendritic cells
CTLs: cytotoxic T lymphocytes
FAS/FASL: fasrymndrit/fasrymndrit ligand
TANs: tumor-associated neutrophils
mIDH1: isocitrate dehydrogenase 1 mutation
TET2: tet methylcytosine dioxygenase 2
IFN: interferon
HLA: human leukocyte antigen
TILs: tumor-infiltrating lymphocytes
shRNA: short hairpin RNA
MVD: microvascular density
HPT: hypofractionated proton therapy
Th1: T helper 1
ERBB2IP: erbb2-interacting protein
MHC: major histocompatibility complex
Tregs: Regulatory T cells
CTLA: cytolytic T lymphocyte-associated antigen
LAIR: leukocyte-associated immunoglobulin-like receptor
EBF1: early B cell factor 1
TLS: tumor lysis syndrome
TNF: tumor necrosis factor
NK: natural killer
ADCC: antibody-dependent cell-mediated cytotoxicity
KIR: killer cell immunoglobulin-like receptor
CXCL: C-X-C motif chemokine ligand
TAA: thioacetamide
TRAIL: tumor necrosis factor-related apoptosis-inducing ligand
GM-CSF: granulocyte-macrophage colony-stimulating factor
IC: immune complexes
TAMs: tumor-associated macrophages
PACT: Prostate cancer associated transcript
ICB: immune checkpoint blockade
LPS: lipopolysaccharide
SCF: stem cell factor
mPFS: median progression free survival
MSI-H: microsatellite instability-high
MMR: mismatch repair
ICI: Immune checkpoint inhibitor
HCC: hepatocellular carcinoma
GEMCIS: Gemcitabine+Cisplatin
CAPOX: Capecitabine+Oxaliplatin
GEMOX: Gemcitabine+Oxaliplatin
FolFox4: Leucovorin+5-fluorouracil+oxaliplatin
EMT: epithelial-mesenchymal transition
